# Effect of *Gymnema montanum* Leaves on Serum and Tissue Lipids in Alloxan Diabetic Rats

**DOI:** 10.1155/EDR.2003.183

**Published:** 2003

**Authors:** R. Ananthan, M. Latha, K. M. Ramkumar, L. Pari, C. Baskar, V. Narmatha Bai

**Affiliations:** 1 Department of Botany Bharathiar University Coimbatore Tamil Nadu India; 2 Department of Biochemistry Faculty of Science Annamalai University Annamalainagar Tamil Nadu 641 046 India

## Abstract

The effect of *Gymnema montanum* leaves on alloxaninduced hyperlipidemia was studied in male Wistar rats. Ethanolic extract of *G. montanum* leaves was administered orally and different doses of the extract on blood glucose, serum and tissue lipids, hexokinase, glucose-6-phosphatase, thiobarbituric acid–reactive substances (TBARS), hydroperoxides, and glutathione in alloxan-induced diabetic rats were studied. *G. montanum* leaf extract (GLEt) at doses of 50, 100, 200 mg/kg body weight for 3 weeks suppressed the elevated blood glucose and lipid levels in diabetic rats. GLEt at 200 mg/kg body weight was found to be comparable to glibenclamide, a reference drug. These data indicate that *G. montanum* represents an effective antihyperglycemic and antihyperlipidemic adjunct for the treatment of diabetes and a potential source of discovery of new orally active agent for future therapy.


*Experimental Diab. Res.*, 4:183–189, 2003
Copyright © Taylor and Francis Inc.
ISSN: 1543-8600 print / 1543-8619 online
DOI: 10.1080/15438600390244353

### Effect of *Gymnema montanum* Leaves on Serum and Tissue Lipids in Alloxan Diabetic Rats

**R. Ananthan,[1] M. Latha,[2] K. M. Ramkumar,[2] L. Pari,[2] C. Baskar,[1] and V. Narmatha Bai[1]**

[1]*Department of Botany, Bharathiar University, Coimbatore, Tamil Nadu, India*
[2]*Department of Biochemistry, Faculty of Science, Annamalai University, Annamalainagar, Tamil Nadu, India*

**The effect of *Gymnema montanum* leaves on alloxan-induced hyperlipidemia was studied in male Wistar rats. Ethanolic extract of *G. montanum* leaves was administered orally and different doses of the extract on blood glucose, serum and tissue lipids, hexokinase, glucose-6-phosphatase, thiobarbituric acid–reactive substances (TBARS), hydroperoxides, and glutathione in alloxan-induced diabetic rats were studied. *G. montanum* leaf extract (GLEt) at doses of 50, 100, 200 mg/kg body weight for 3 weeks suppressed the elevated blood glucose and lipid levels in diabetic rats. GLEt at 200 mg/kg body weight was found to be comparable to glibenclamide, a reference drug. These data indicate that *G. montanum* represents an effective antihyperglycemic and antihyperlipidemic adjunct for the treatment of diabetes and a potential source of discovery of new orally active agent for future therapy.**

**Keywords** Alloxan Diabetes; Blood Glucose; Carbohydrate Enzymes; *Gymnema montanum*; Lipids

Experimental diabetes in animals has provided considerable insight into the physiologic and biochemical derangements of the diabetic state. Many of the derangements have been characterized in hyperglycemic animals. Significant changes in lipid metabolism and structure also occur in diabetes [1]. In these cases, the structural changes are clearly oxidative in nature and are associated with development of vascular disease in diabetes [2]. In diabetic rats, increased lipid peroxidation was also associated with the hyperlipidemia [3]. Liver, an insulin-dependent tissue that plays a pivotal role in glucose and lipid homeostasis, is severely affected during diabetes [4]. Liver participates in the uptake, oxidation, and metabolic conversion of free fatty acids, synthesis of cholesterol, phospholipids, and triglycerides. During diabetes, a profound alteration in the concentration and composition of lipid occurs. Decreased glycolysis, impeded glycogenesis, and increased glyconeogenesis are some of the changes of glucose metabolism in the diabetic liver [5].

For various reasons, in recent years the popularity of complementary medicine has increased. Dietary measures and traditional plant therapies as prescribed by ayurvedic and other indigenous systems of medicine are used commonly in India [6]. In recent times, many traditionally used medicinally important plants were tested for their antidiabetic potential by various investigations in experimental animals [7].

*Gymnema montanum* Hook [8] belongs to the family Asclepiadaceae; it is an endemic and endangered plant species of India found mainly in Western ghats [9]. Except a few common species, the majority of the species shows various levels of rarity and *G. montanum* is one such species that have been taken for our study.

Previously we have reported the effect of *G. montanum* on rate-limiting enzymes of glycolysis and gluconeogenesis in liver of diabetic animals [10]. This study was thus initiated with the aim of evaluating the effects of ethanolic extract of *G. montanum* leaves on serum and tissue lipids in alloxan diabetic rats.

Received 31 March 2003; accepted 17 July 2003.
Address correspondence to R. Ananthan, Department of Botany, Bharathiar University, Coimbatore-641 046, Tamilnadu, India. E-mail: botananthan@lycos.com

### MATERIALS AND METHODS

### Animals

Male albino Wistar rats, body weight of 180 to 200 g, bred in Central Animal House, Rajah Muthiah Medical College, Annamalai University, were used in this study. The animals were fed on a pellet diet (Hindustan Lever, India) and water ad libitum. The animals were maintained in a controlled environment under standard conditions of temperature and humidity with an alternating light and dark cycle. The animals used in the present study were maintained in accordance with the guidelines prescribed by the National Institute of Nutrition, Indian Council of Medical Research, Hyderabad, India, and approved by the Ethical Committee, Annamalai University.

### Plant Material

*G. montanum* leaves were collected freshly from the Shola forests of Western Ghats, Gudalur, The Nilgiri Biosphere Reserve, at an altitude of 900 to 1500 m above sea level. The plant was identified at the Herbarium of Botanical Survey of India, Southern Circle, Coimbatore, India (accession no. 32561-65) and was deposited in the Department of Botany, Bharathiar University.

### Preparation of Plant Extract

To prepare the *G. montanum* leaf extract (GLEt), 500 g of fresh leaves of *G. montanum* were chopped into small pieces and soaked overnight in 1.5 L of 95% ethanol. This suspension was filtered and the residue was resuspended in an equal volume of 95% ethanol for 48 hours and filtered again. The two filtrates were pooled and the solvents were evaporated in a rotavapor at 40°C to 50°C under reduced pressure and lyophilized. A greenish-black powdered material was obtained (20 to 30 g). It was stored at 0°C to 4°C until used. When needed, the residual extract was suspended in distilled water and administered by oral intragastric tubes [11].

### Experimental Induction of Diabetes

Rats were injected with freshly prepared solution of alloxan monohydrate in normal saline by intraperitoneally at a dose of 150 mg/kg body weight [12]. Because alloxan is capable of producing fatal hypoglycemia as a result of massive pancreatic insulin release, rats were treated with 20% glucose solution (5 to 10 mL) orally after 6 hours. The rats were then kept for the next 24 hours on 5% glucose solution bottles in their cages to prevent hypoglycemia [13]. After 3 weeks, rats with moderate diabetes having glycosuria and hyperglycemia (i.e., with blood glucose of 200 to 300 mg/dL) were chosen for the experiment.

### Experimental Procedure

A total of 42 rats (30 diabetic surviving rats, 12 normal rats) were used and they were divided into 7 groups of 6 rats each.

Group 1: Normal untreated rats.
Group 2: Normal rats treated with GLEt (200 mg/kg body weight [b.w.])
Group 3: Diabetic control.
Group 4: Diabetic rats treated with GLEt (50 mg/kg b.w.)
Group 5: Diabetic rats treated with GLEt (100 mg/kg b.w.)
Group 6: Diabetic rats treated with GLEt (200 mg/kg b.w.)
Group 7: Diabetic rats treated with glibenclamide (600 $\mu$g/kg b.w.) [14].

The GLEt and the reference drug glibenclamide were administered by oral intragastric tubes during the experimental period. Body weights and food and fluid intake of the experimental animals were recorded at regular intervals. Food intake was determined by measuring the difference between the preweighed standard food and weight of the balanced and spilled food for every 24 hours. Similarly the fluid intake was measured by monitoring the amount of fluid given and the balanced fluid.

At the end of 3 weeks, the animals were deprived of food overnight and sacrificed by decapitation. Blood was taken from the jugular vein and collected in 2 tubes: one with potassium oxalate and sodium fluoride solution for plasma and another without anticoagulant for serum separation. The liver was immediately dissected out, washed in ice-cold saline, patted dry, and weighed.

### Analytical Methods

Blood glucose was determined by the *o*-toluidine method [15]. Plasma insulin was assayed by enzyme-linked immunosorbent assay (ELISA) method, using Boeheringer-Mannheim Kit with a Boeheringer analyzer ES300 using human insulin as standard [16].

Lipids were extracted from serum and tissues by the method of Folch and colleagues [17]. Total cholesterol and triglycerides were estimated by the method of Zlatkis and colleagues [18] and Foster and Dunn [19], respectively. Free fatty acids and phospholipids were analysed by the method of Falholt and colleagues [20] and Zilversmit and Davis [21]. The extent of lipid peroxidation was estimated colorimetrically by thiobarbituric acid–reactive substances (TBARS) and hydroperoxides by the method of Nichans and Samuelson [22] and Jiang and colleagues [23], respectively. Reduced glutathione (GSH) was determined by the method of Ellman [24]. Hexokinase (E.C 2.7.1.1) and glucose-6-phosphatase (E.C 3.1.3.9) were assayed according to the method of Brandstrup and colleagues [25] and Koida and Oda [26], respectively.

### Statistical Analysis

All the data were expressed as mean ± SD of number of experiments (n = 6). The statistical significance was evaluated by one-way analysis of variance (ANOVA) using SPSS version 7.5 (SPSS, Cary, NC, USA) and the individual comparisons were obtained by Duncan's multiple range test (DMRT) [27].

### RESULTS

The results of the present investigation are depicted in Figure 1 and Tables 1 to 4. The treatment with ethanolic extract of *G. montanum* leaves reversed the weight loss in diabetic rats.

The body weight changes of the experimental animals are presented in Figure 1. At the end of the experimental period, the body weights of the diabetic animals were significantly reduced when compare to normal animals. The body weights in the GLEt-treated and glibenclamide-treated groups were increased significantly ($P < .001$) at the end of the 3rd week when compared with the diabetic control group.

Table 1 represents the changes in the water and food intake in normal and experimental diabetic rats. The food and water intake significantly increased in diabetic control rats and it was significantly reduced in GLEt- and glibenclamide-treated groups than diabetic control rats.

In all groups prior to alloxan administration, the basal levels of blood glucose of the rats were not significantly different. However, 15 days after alloxan administration, blood glucose levels were significantly higher in the experimental rats selected for the study. Table 2 shows the effect of GLEt and glibenclamide on blood glucose levels. Although a significant antihyperglycemic effect was evident from the 1st week onwards, the decrease in blood glucose was maximum on 3rd week, with significant increase in plasma insulin in group receiving 200 mg/kg body weight of GLEt. The effect of GLEt at

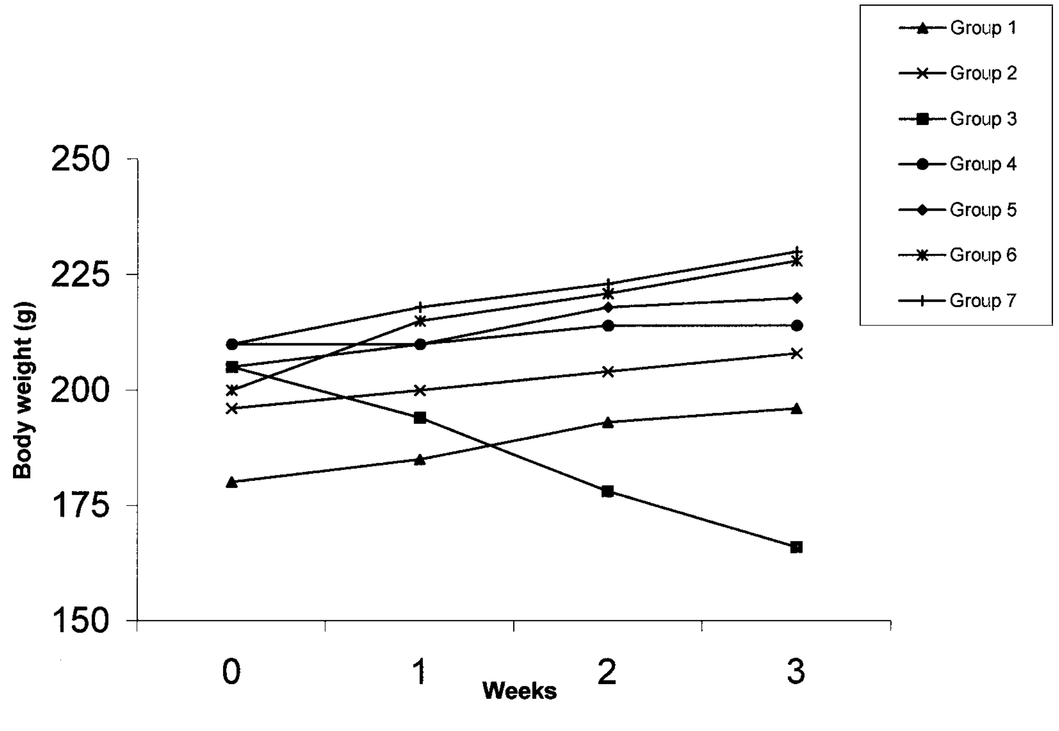

**FIGURE 1**
Body weights (g) of control and experimental rats (mean ± SD; n = 6). Group 1, normal untreated rats; group 2, normal rats given GLEt (200 mg $kg^{-1}$ b.w.); group 3, diabetic control; group 4, diabetic rats given GLEt (50 mg $kg^{-1}$ b.w.); group 5, diabetic rats given GLEt (100 mg $kg^{-1}$ b.w.); group 6, diabetic rats given GLEt (200 mg $kg^{-1}$ b.w.); group 7, diabetic rats given glibenclamide (600 mg $kg^{-1}$ b.w.). Measurements were taken weekly until the experimental period. b.w. = body weight.

**TABLE 1**
Changes in food intake and fluid intake in normal and experimental rats

| Groups | Fluid intake (mL/rat per day) | | Food intake (g/rat per day) | |
|---|---|---|---|---|
| | Before | After | Before | After |
| Normal | 75 ± 8.12 | 73 ± 10[a] | 15 ± 0.8 | 14 ± 0.9[a] |
| Normal + GLEt (200 mg/kg) | 80 ± 6.07 | 77 ± 3[a] | 18 ± 0.9 | 16 ± 0.8[a] |
| Diabetic control | 155 ± 17.0 | 165 ± 30[b] | 40 ± 3.0 | 56 ± 7.0[b] |
| Diabetic + GLEt (50 mg/kg) | 135 ± 12.0 | 100 ± 15[c] | 35 ± 5.0 | 48 ± 9.3[c] |
| Diabetic + GLEt (100 mg/kg) | 128 ± 10.3 | 95 ± 9[d] | 32 ± 6.2 | 40 ± 6.5[d] |
| Diabetic + GLEt (200 mg/kg) | 120 ± 9.85 | 80 ± 7[e] | 25 ± 2.0 | 30 ± 1.2[e] |
| Diabetic + glibenclamide | 126 ± 22.0 | 92 ± 18[f] | 25 ± 1.5 | 32 ± 2.0[e] |

*Note.* Values are given as mean ± SD from 6 rats in each group.
Values not sharing a common superscript letter differ significantly at $P < .05$ (DMRT), Duncan procedure; range for the levels 2.95, 3.09, 3.20.
Superscripts denote significance.

a dose of 200 mg/kg body weight was significant as compared to 50 and 100 mg/kg body weight and therefore the higher dose was used for further biochemical studies. Normal rats treated with 200 mg/kg body weight of GLEt also showed a decrease in blood glucose level.

The effect of GLEt on serum and tissue lipids of normal and experimental rats is summarized in Table 3. A marked increase in the frequency of cholesterol, free fatty acids, triglycerides, and phospholipids were observed in diabetic control rats. Treatment with GLEt significantly reduced the lipid levels.

The extent of lipid peroxidation, glutathione content, and the activities of carbohydrate enzymes are represented in Table 4. TBARS, hydroperoxides, and glucose-6-phosphatase activity were significantly increased, whereas glutathione and hexokinase activities were significantly decreased in liver of diabetic rats. Treatment with GLEt significantly increased the glutathione level and hexokinase activities and reduced the level of TBARS, hydroperoxides, and glucose-6-phosphatase activity. Normal rats treated with GLEt showed significant changes in tissue lipids.

**TABLE 2**
Changes in blood glucose and plasma insulin of normal and experimental animals

| Groups | Blood glucose (mg/dL) | | | | | Plasma insulin ($\mu$U/mL) after 3 weeks |
|---|---|---|---|---|---|---|
| | Day 0 | 15 days after alloxan injection | 1 week after treatment | 2 weeks after treatment | 3 weeks after treatment | |
| Normal | 79 ± 3.03 | 84 ± 5.14 | 82 ± 5.92 | 80 ± 6.04 | 81 ± 5.98 | 13.62 ± 5.52[a] |
| Normal + GLEt (200 mg/kg) | 84 ± 5.59 | 83 ± 4.66 | 81 ± 5.56 | 79 ± 4.30 | 75 ± 4.99 | 15.13 ± 0.90[b] |
| Diabetic control | 81 ± 4.94 | 265 ± 19.45** | 279 ± 12.93** | 285 ± 12.95** | 298 ± 15.77** | 5.50 ± 2.75[c] |
| Diabetic + GLEt (100 mg/kg) | 83 ± 6.59 | 248 ± 14.02 | 215 ± 13.04* | 180 ± 9.83** | 161 ± 13.25** | 9.16 ± 0.62[d] |
| Diabetic + GLEt (200 mg/kg) | 79 ± 5.09 | 258 ± 18.61 | 200 ± 12.58** | 120 ± 6.39** | 86 ± 9.25** | 21.06 ± 4.32[e] |
| Diabetic + glibenclamide (600 $\mu$g/kg) | 77 ± 4.48 | 245 ± 13.99 | 219 ± 7.05* | 191 ± 10.8** | 118 ± 4.48** | 10.08 ± 3.13[f] |

*Note.* Values are given as mean ± SD for 6 rats in each group.
Diabetic control was compared with normal. Experimental groups were compared with corresponding values after alloxan injection (15 days). $^{*}P < .01$, $^{**}P < .001$.
Values not sharing a common superscript letter differ significantly at $P < .05$ (DMRT), Duncan procedure; range for the levels 2.89, 3.03, 3.13, 3.20, 3.25.

**TABLE 3**

Changes in levels of cholesterol, free fatty acid, triglycerides, and phospholipids in serum and liver of normal and experimental animals

| Groups | Cholesterol | | Free fatty acid | | Triglycerides | | Phospholipids | |
|---|---|---|---|---|---|---|---|---|
| | Liver (mg/100 g wet tissue) | Serum (mg/dL) | Liver (mg/100 g wet tissue) | Serum ($\mu$M/L) | Liver (mg/100 g wet tissue) | Serum (mg/dL) | Liver (g/100 g wet tissue) | Serum (mg/dL) |
| Normal | $330 \pm 2.62^a$ | $77 \pm 3.41^a$ | $606 \pm 3.98^a$ | $2757 \pm 342.0^a$ | $341 \pm 4.81^a$ | $42 \pm 3.69^a$ | $1.6 \pm 0.04^a$ | $81 \pm 4.99^a$ |
| Normal + GLEt (200 mg/kg) | $325 \pm 4.02^a$ | $70 \pm 2.16^b$ | $580 \pm 5.21^a$ | $2379 \pm 277.1^b$ | $331 \pm 4.29^a$ | $39 \pm 1.62^a$ | $1.54 \pm 0.05^a$ | $76 \pm 2.62^b$ |
| Diabetic control | $513 \pm 4.57^b$ | $95 \pm 7.51^c$ | $911 \pm 8.29^b$ | $3366 \pm 727.3^c$ | $620 \pm 8.79^b$ | $67 \pm 5.77^b$ | $2.93 \pm 0.28^b$ | $102 \pm 8.70^c$ |
| Diabetic + GLEt (200 mg/kg) | $417 \pm 4.11^c$ | $81 \pm 4.30^d$ | $769 \pm 3.11^c$ | $3030 \pm 417.0^d$ | $461 \pm 5.89^c$ | $50 \pm 4.20^c$ | $1.97 \pm 5.58^c$ | $87 \pm 5.05^d$ |
| Diabetic + glibenclamide (600 $\mu$g/kg) | $438 \pm 4.87^d$ | $90 \pm 4.86^e$ | $800 \pm 6.17^d$ | $3124 \pm 611.4^e$ | $532 \pm 6.35^d$ | $59 \pm 5.10^d$ | $2.11 \pm 0.19^d$ | $91 \pm 5.80^e$ |

*Note.* Values are given as mean ± SD for 6 rats in each group.
Values not sharing a common superscript letter differ significantly at $P < .05$ (DMRT), Duncan procedure; range for the levels 2.95, 3.09, 3.20.

**TABLE 4**
Change in the levels of liver TBARS, hydroperoxides, glutathione, hexokinase, and glucose-6-phosphatase in normal and experimental animals

| Groups | TBARS (mM/100 g tissue) | Hydroperoxides (mM/100 g tissue) | Glutathione (mg/100 mg tissue) | Hexokinase (units*/g protein) | Glucose-6-phosphatase (units**/mg protein) |
|---|---|---|---|---|---|
| Normal | $0.76 \pm 0.02^{a}$ | $75 \pm 3.96^{a}$ | $46 \pm 3.9^{a}$ | $142 \pm 4.89^{a}$ | $0.162 \pm 0.01^{a}$ |
| Normal + GLEt (200 mg/kg) | $0.69 \pm 0.02^{b}$ | $68 \pm 2.12^{b}$ | $53 \pm 2.02^{b}$ | $151 \pm 3.07^{b}$ | $0.158 \pm 0.01^{a}$ |
| Diabetic control | $1.72 \pm 0.10^{c}$ | $93 \pm 7.66^{c}$ | $23 \pm 1.86^{c}$ | $109 \pm 4.71^{c}$ | $0.272 \pm 0.021^{b}$ |
| Diabetic + GLEt (200 mg/kg) | $1.23 \pm 0.03^{d}$ | $78 \pm 5.58^{d}$ | $40 \pm 2.88^{d}$ | $139 \pm 8.09^{d}$ | $0.171 \pm 0.024^{a,c}$ |
| Diabetic + glibenclamide (600 $\mu$g/kg) | $1.30 \pm 0.04^{e}$ | $81 \pm 4.52^{d}$ | $34 \pm 2.91^{e}$ | $137 \pm 9.40^{d}$ | $0.182 \pm 0.020^{c}$ |

*Note.* Values are given as mean ± SD for 6 rats in each group.
Values not sharing a common superscript letter differ significantly at $P < .05$ (DMRT), Duncan procedure; range for the levels 2.95, 3.09, 3.20.
*$\mu$moles of glucose phosphorylated/min.
**$\mu$moles of Pi liberated/min.

### DISCUSSION AND CONCLUSION

Hyperlipidemia is a metabolic complication of both clinical and experimental diabetes [28]. In animals, the administration of diabetogenic doses of alloxan induces hyperlipidemia [29]. In our present study, we have observed that an ethanolic extract of GLEt can reverse the effects. The possible mechanism by which GLEt brings about its antihyperglycemic action may be by potentiation of pancreatic secretion of insulin from beta cells of islets or due to enhanced transport of blood glucose to peripheral tissue. This was clearly evidenced by the increased levels of insulin in diabetic rats treated with GLEt. In this context, a number of other plants have also been reported to have antihyperglycemic and insulin-release stimulatory effect [30, 31].

Excess free fatty acids in serum produced by the alloxan lowers the insulin-mediated glucose disposal and promotes conversion of excess fatty acids into phospholipids and cholesterol in liver. These two substances along with excess triglycerides formed at the same time in the liver may be discharged into the blood in the form of lipoprotein [32]. Both increased hepatic production of triglycerides and decreased peripheral removal have been demonstrated. Hypercholesteremia and hypertriglyceridemia have been reported to occur in diabetic rats [33]. A high concentration of cholesterol in human serum is one of the primary factors in the development of atherosclerosis [34]. The marked hyperlipidemia that characterizes the diabetic state may therefore be regarded as a consequence of the uninhibited actions of lipolytic hormones on the fat depot [35].

The antihyperlipidemic effect of GLEt may be due to the down-regulation of NADPH and NADH, cofactors in the fat metabolism. Higher activity of glucose-6-posphatase provides $H^+$ that binds with $NADP^+$ to form of NADPH and is helpful in the synthesis of fats from carbohydrates. When glycolysis slows down because of cellular activity, the pentose phosphate pathway still remains active in liver to breakdown glucose, which continuously provides NADPH. Enhanced hexokinase activity in GLEt-treated rats suggests greater uptake of glucose from blood by the liver cells.

Activities of enzyme suggest that enhanced lipid metabolism during diabetes is shifted toward carbohydrate metabolism and it enhances the utilization of glucose at the peripheral sites. One of the possible actions of GLEt may be due to its inhibition on endogenous synthesis of lipids.

Alteration of fatty acid composition by increased lipid levels contribute to lowering the resistance of tissues and higher rate of oxidative stress. Our results show increased lipid peroxidation markers TBARS and hydroperoxides contributing to oxidative stress. Decreased activity of glucose-6-phosphatase through pentose phosphate shunt results in a high reduced glutathione to oxidized glutathione ratio (GSH/GSSG) [32], which is coupled with the conversion of NADPH to NADP. Administration of GLEt to diabetic rats decreased lipid peroxidation and increased the glutathione content. GLEt may produce high levels of $NADP^+$, which result in down-regulation of lipogenesis and lower risk of the tissues for oxidative stress and higher resistance for diabetes.

The overwhelming evidence demonstrated above indicates that hyperglycemia coupled with hyperlipidemia increases the risk for cardiovascular diseases. GLEt significantly reduces the levels of serum and tissue lipids, which are actively raised in alloxan diabetic rats. GLEt has beneficial effect on plasma insulin and hexokinase activity. These findings strengthen the observation that naturally occurring compounds of plant origin have antidiabetic effects.

### REFERENCES

[1] Sochar, M., Baquer, N. Z., and Mclean, P. (1985) Glucose under utilization in diabetes. Comparative studies on the changes in the activities of enzymes of glucose metabolism in rat kidney and liver. *Mol. Physiol.,* **7,** 51–68.

[2] Baynes, J. W., and Thrope, S. R. (1999) Role of oxidative stress in diabetic complications. *Diabetes,* **48,** 1–4.

[3] Morel, D. W., and Chisolm, G. M. (1989) Antioxidant treatment of diabetic rats inhibits lipoprotein oxidation and cyto toxicity. *J. Lipid Res.,* **30,** 1827–1834.

[4] Seifter, S., and England, S. (1982) Energy metabolism. In: *The Liver: Biology and pathobiology*, Edited by Arias, I., Papper, M., Schacter, D. et al., pp. 219–249, New York, Raven Press.

[5] Baquer, N. Z. (1998) Glucose over utilization and under utilization in diabetes and effects of antidiabetic compounds. *Ann. Real Acad. Farm.,* **64,** 147–180.

[6] Warier, P. K. (1995) *Eugenia jambolana* Linn. In: *Indian Medicinal Plants*. Edited by Warier, P. K., Nambiar, V. P. K., and Ramankutty, C., pp. 48–51, Madras, Orient Longman.

[7] Srivastava, Y., Bhat, H. V., Verma, Y., and Venkaidh, K. (1993) Antidiabetic and adaptogenic properties of *Momordica charantia* extract: An experimental and clinical evaluation. *Phytother. Res.,* **7,** 285–289.

[8] Hooker, J. D. (1883) Asclepiadaceae. In: *Hookers' Flora of British India*, vol. 4, pp. 31. Dehradun, India, International Book Distributors.

[9] Vajravelu, E., and Bhargavan, P. (1983) Rare and endemic species recollected after fifty years or more from South India. An assessment of threatened plants of India. In: *Proceedings of the Seminar Held at Dehradun*. Edited by Jain, S. K., and Raw, R. R., pp. 14–17. BSI, Botanic Garden, HowRah.

[10] Ananthan, R., Latha, M., Ramkumar, K. M., Baskar, C., and Narmatha Bai, V. (2003) Effect of *Gymnema montanum* on blood glucose, plasma insulin and carbohydrate metabolic enzymes in alloxan induced diabetic rats. *J. Med. Food.,* **6,** 43–49.

[11] Hossain, M. Z., Shibib, B. A., and Rahman, R. (1992) Hypoglycemic effects of *Coccinia indica*: Inhibition of key gluconeogenic enzyme, glucose-6-phosphatase. *Ind. J. Exp. Biol.,* **10,** 418–420.

[12] Al-Shamaony, L., Al-Khazraji, S. M., and Twaiji, H. A. (1994) Hypoglycemic effect of *Artemisia herba alba* II. Effect of a valuable extract on some blood parameters in diabetic animals. *J. Ethnopharmacol.,* **43,** 167–171.

[13] Gupta, M. P., Solis, N. G., Esposito, M., and Sanchez, S. (1984) Hypoglycemic activity of *Neurolaena lobata* (L) R. Br. *J. Ethnopharmacol.,* **10,** 323–327.

[14] Pari, L., and Uma Maheswari, J. (2000) Antihyperglycemic activity of *Musa sapientum* flowers: Effect on lipid peroxidation in alloxan diabetic rats. *Phytother. Res.,* **14,** 1–3.

[15] Sasaki, T., Masty, S., and Sonae, A. (1972) Effect of acetic acid concentration on the colour reaction in the *O*-toluidine boric acid method for blood glucose estimation. *Rinshbo Kagaku,* **1,** 346–353.

[16] Anderson, L., Dinesen, B., Jorgensen, P. N., Poulsen, F., and Roder, M. F. (1993) Enzyme immunoassay for intact human insulin in serum or plasma. *Clin. Chem.,* **38,** 578.

[17] Folch, J., Less, M., and Solane, S. G. H. (1957) A simple method for isolation and purification of total lipids from animal tissues. *J. Biol. Chem.,* **26,** 497–509.

[18] Zlatkis, A., Zak, B., and Bogle, G. J. (1953) A method for the determination of serum cholesterol. *J. Clin. Med.,* **41,** 486–492.

[19] Foster, L. B., and Dunn, R. T. (1973) Stable reagents for determination of serum triglycerides by colorimetric hantzsch condensation method. *Clin. Chem.,* **19,** 338–340.

[20] Falholt, K., Falholt, W., and Lund, B. (1973) An easy colorimetric method for routine determination of free fatty acids in plasma. *Chem. Acta.,* **46,** 105–111.

[21] Zilversmit, D. B., and Davis, A. K. (1950) Micro determination of phospholipids by TCA precipitation. *J. Lab. Clin. Med.,* **35,** 155–161.

[22] Nichans, W. G., and Samuelson, D. (1968) Formation of malodialdehyde from phospholipid arachidonate during microsomal lipid peroxidation. *Eur. J. Biochem.,* **6,** 126–130.

[23] Jiang, Z. Y., Hunt, J. V., and Wolff, S. P. (1992) Ferrous ion oxidation in the presence of xylenol orange for detection of lipid hydroperoxide in low-density lipoprotein. *Anal. Biochem.,* **202,** 384–389.

[24] Ellman, G. L. (1959) Tissue sulfhydryl groups. *Arch. Biochem. Biophys.,* **82,** 70–77.

[25] Brandstrup, N., Kirk, J. E., and Bruni, C. (1957) Determination of hexokinase in tissues. *J. Gerontol.,* **12,** 166–171.

[26] Koida, H., and Oda, T. (1959) Pathological occurrence of glucose-6-phosphatase in liver disease. *Clin. Chem. Acta.,* **4,** 554–561.

[27] Duncan, B. D. (1957) Multiple range tests for correlated and heteroscedastic means. *Biometrics,* **13,** 359–364.

[28] Bierman, E. L., Amaral, J. A. P., and Balknap, B. H. (1975) Hyperlipemia and diabetes mellitus. *Diabetes,* **25,** 509–515.

[29] Velminsky, J., Burr, I. M., and Stauffacher, W. (1970) Comparative study of early metabolic events resulting from the administration of the two diabetogenic agents alloxan and streptozotocin. *Eur. J. Clin. Invest.,* **1,** 104–108.

[30] Latha, M., and Pari, L. (2003) Preventive effects of *Cassia auriculata* L. flowers on brain lipid peroxidation in rats treated with streptozotocin, *Mol. Cell Biochem.,* **243,** 23–28.

[31] Venkateswaran, S., and Pari, L. (2002) Effect of *Coccinia indica* on blood glucose, insulin and hepatic key enzymes in experimental diabetes. *Pharmacol. Biol.,* **40,** 165–170.

[32] Bopanna, K. N., Kannan, J., Sushma, G., Balaraman, R., and Rathod, S. P. (1997) Antidiabetic and antihyperlipidemic effects of neem seed kernel powderonalloxan diabetic rabbits. *Ind. J. Pharmacol.,* **29,** 162–167.

[33] Pushparaj, P., Tan, C. H., and Tan, B. K. H. (2000) Effects of *Averrhoa bilimli* leaf extract on blood glucose and lipids in streptozotocin diabetic rats. *J. Ethnopharmacol.,* **72,** 69–76.

[34] Milagro, F. I., and Martinez, J. A. (2000) Effects of oral administration of a $\beta_3$-adrenergic agonist on lipid metabolism in alloxan diabetic rats. *J. Pharm. Pharmacol.,* **52,** 851–856.

[35] Goodman, L. S., and Gilman, A. (1985) *The Pharmacological Basis of Therapeutics,* 7th ed., pp. 1490–1510. New York, MacMillan.

